# Evaluation of an electricity-independent method for IS*2404* Loop-mediated isothermal amplification (LAMP) diagnosis of Buruli ulcer in resource-limited settings

**DOI:** 10.1371/journal.pntd.0012338

**Published:** 2024-08-14

**Authors:** Evans K. Ahortor, Theresa Manful Gwira, Samiratu Mahazu, Astrid C. Erber, Anthony Ablordey

**Affiliations:** 1 Department of Bacteriology, Noguchi Memorial Institute for Medical Research, University of Ghana, Legon, Ghana; 2 School of Pharmacy and Pharmaceutical Science, Cardiff University, Cardiff, Wales, United Kingdom; 3 Department of Biochemistry, Cell and Molecular Biology & West African Centre for Cell Biology of Infectious Pathogens, School of Biological Sciences, College of Basic and Applied Sciences, University of Ghana, Legon, Ghana; 4 Department of Molecular Microbiology and Immunology, Tokyo Medical and Dental University, Tokyo, Japan; 5 Department of Epidemiology, Centre for Public Health, Medical University of Vienna, Vienna, Austria; 6 Infectious Diseases Data Observatory, Oxford, United Kingdom; Johns Hopkins University, UNITED STATES OF AMERICA

## Abstract

**Introduction:**

Buruli ulcer (BU) caused by *Mycobacterium ulcerans* (MU) is a devastating necrotic skin disease. PCR, recommended for confirmation of BU by WHO, requires an adequately equipped laboratory, therefore often delaying timely diagnosis and treatment of BU patients in remote settings. Loop-mediated isothermal amplification (LAMP) is a PCR-based protocol for isothermal amplification of DNA that has been suggested for diagnosis of BU in low-resource settings.

**Study aims and methods:**

This is an exploratory diagnostic test evaluation study, with an embedded qualitative sub-study. Its aims are two-fold: First, to evaluate a simple rapid syringe-based DNA extraction method (SM) in comparison with a more elaborate conventional DNA extraction method (CM), followed by a LAMP assay targeting IS*2404* for the detection of MU, either using a commercially available pocket warmer (pw) or a heat block (hb) for incubation. Second, to complement this by exploring the diagnostic workflow for BU at a community-based health centre in an endemic area in rural Ghana as an example of a potential target setting, using interviews with researchers and health care workers (HCWs). Diagnostic test evaluation results are discussed in relation to the requirements of a target product profile (TPP) for BU diagnosis and the target setting.

**Results:**

A protocol using SM for DNA extraction followed by IS*2404* PCR (IS*2404* PCR^SM^) was able to identify MU DNA in 73 out of 83 BU clinical specimens submitted for diagnosis. The sensitivity, specificity, positive predictive value (PPV) and negative predictive value (NPV) of IS*2404* PCR^SM^ were 90.12%, 100%, 100% and 65.21% respectively, as compared to the reference standard IS*2404* PCR in combination with a standard extraction protocol for mycobacterial DNA. Evaluation of the LAMP assay on 64 SM DNA extracts showed a sensitivity, specificity, PPV and NPV of 83.6%, 100%, 100% and 50%, respectively, using either pocket warmer (pwLAMP^SM^) or heat block (hbLAMP^SM^) for incubation of the reaction, as compared to the same reference standard. The limit of detection of pwLAMP^SM^ was found to be 30 copies of the IS*2404* target. Interview findings explored barriers to BU diagnosis and treatment, including perceptions of the disease, costs, and availability of transport. Participants confirmed that a diagnosis at the PoC, in addition to screening based on clinical criteria, would be advantageous in order to prevent delays and loss to follow-up.

**Discussion and conclusions:**

The high diagnostic and analytic accuracy of the pwLAMP, evaluated by us in combination with a syringe-based DNA extraction method, supports its potential use for the rapid detection of MU in suspected BU samples at the community or primary health care level without reliable electricity supply. Further optimization needs include a lysis buffer, evaluation directly at the PoC and/or other sites, assessing staff training requirements and quality control.

## Author summary

Buruli ulcer (BU) is a severe bacterial skin disease. Molecular diagnostic methods, as recommended by the World Health Organization (WHO), require a well-equipped laboratory with reliable electricity supply, which is challenging in low-resource settings. We aimed to evaluate a protocol for loop-mediated isothermal amplification (LAMP), independent of power supply, as an alternative DNA-detection based diagnostic tool for peripheral health care facilities at the point-of-care (PoC). Our protocol included a rapid syringe-based DNA extraction method, followed by a LAMP assay using a commercial pocket warmer for incubation. It showed promising results, with around 84% sensitivity and 100% specificity, as compared to standard molecular methods. Through interviews and discussions with researchers, healthcare workers and community members, we characterized the diagnostic and treatment workflow at a potential target site in an endemic area in Ghana’s Eastern Region. Reported barriers to treatment seeking included perceptions of BU and price and availability of transport, and confirmed potential advantages of diagnosis at the PoC in preventing delays and loss to follow-up. Our study suggests that this LAMP protocol could hold promise for rapid and accurate diagnosis of BU in settings with unreliable electricity; further research will need to include further optimization and evaluation.

## Introduction

Buruli ulcer (BU) disease caused by *Mycobacterium ulcerans* (MU) is the third most common tropical mycobacterial pathogen after tuberculosis and leprosy [1]. BU is mainly an infection of the skin that leads to extensive skin damage, as observed in most BU patients [2–4]. Skin destruction is caused by the production of a polyketide-derived macrolide called mycolactone, a lipid toxin produced by MU [5,6]. The disease, reported in about 33 countries worldwide [7–9], is a major public health problem in several rural communities in the West African countries of Ivory Coast, Ghana and Benin [10–14]. A major challenge for the control of BU is the lack of rapid detection tools. Mycobacterial culture is considered the gold standard for BU diagnosis in terms of sensitivity and specificity; however, this method cannot be relied upon for timely treatment of patients because it takes between 6–8 weeks for the pathogen to grow on artificial media. Histopathology once served as a useful method for BU diagnosis from biopsies. With the current recommended sampling methods fine needle aspirates (FNAs) and swabs, the usefulness of this method has diminished [15]. Additionally, there are no approved immunological diagnostic tools for the detection of MU, although several studies have sought to identify a unique and specific biomarker for BU diagnosis [1,16]. Nausch and colleagues found specific CD4+ T cells producing TNFα to be associated with lesion size and healing progress; the potential of these to serve as diagnostic markers is to be investigated [1]. The preferred confirmatory test for BU diagnosis is the polymerase chain reaction (PCR). The most commonly used PCR protocol targets the repetitive insertion sequence (IS) *2404* occurring more than 200 times within the genome of *M*. *ulcerans* [17]. IS*2404* PCR has a sensitivity between 92 and 95% [18,19].

A major limitation of PCR is its requirement for lengthy DNA preparation steps and equipment needs that most often can’t be accessed at the primary health-care level. Development of a field-applicable diagnostic tool for rapid detection of MU continues to be one of the important research priorities outlined by the WHO’s Global BU initiative [20]. Thus, there is the need to develop a tool paralleling PCR in terms of sensitivity and specificity that can be applied at the primary and community-level for point-of-care (PoC) diagnosis of BU in remote areas. Loop-Mediated Isothermal Amplification (LAMP) [21–24] is an innovative isothermal DNA amplification technique which has been developed to accelerate the detection of DNA without the need of sophisticated DNA amplification instruments like a thermal cycler. The LAMP readout method is simply a change in colour of the reaction mixture. Thus, LAMP has an added advantage over PCR techniques in that it does not require expensive PCR equipment and a gel-imaging step to visualize the amplicons. LAMP has been applied for the detection of clinically relevant pathogens [25–29], including MU [30–34]. Lyophilized LAMP reagents have been investigated for BU [32] and other diseases [35] to confer stability, in particular under elevated temperature conditions. In our previous publication [30], we explored the use of crude DNA extracts as template for the LAMP assay and a disposable pocket warmer as innovative means of achieving an isothermal condition to support the LAMP assay. We observed that the sensitivity of the pocket warmer LAMP (pwLAMP) assay was low when crude rather than pure DNA extracts (using commercial kits) were used [30]. However, preparing purified DNA requires the use of expensive DNA extraction kits and auxiliary equipment such as centrifuges.

The aims of this exploratory study are two-fold. First, we investigated the suitability of a hand-operated, syringe-based device to extract DNA of sufficient purity from BU clinical specimens, and subsequently amplified and detected this DNA using LAMP targeting the IS*2404* sequence, using a commercial pocket warmer for incubation. The syringe device, operated manually, does not require a centrifuge and is therefore, like the pocket warmer, independent of electricity supply. We discuss our suggested LAMP protocol in relation to the requirements of a target product profile (TPP) for BU diagnosis at the PoC [36]. Second, in order to contextualize our research, we characterized a potential target setting for BU diagnosis using LAMP, a community-based primary health care centre in an endemic area Ghana’s Eastern Region. We were interested in the conditions at the site, as well as the diagnostic and treatment workflow including the challenges BU patients had to meet.

## Materials and methods

### Ethics statement

Archived samples used for our previous study [30] were used for this study. Ethical clearance for the use of these samples was obtained from the Institutional Review Board (IRB) of the Noguchi Memorial Institute for Medical Research, University of Ghana (Certified Protocol Number CPN 026/10-11). Patients were given detailed information about the use of their samples, and written consent was obtained from them or, in case of minors, their parents.

For the interviews and focus group discussion (FGD), the study protocol was approved by the IRB of the Noguchi Memorial Institute for Medical Research, University of Ghana (Certified Protocol Number CPN 026/10-11 amend 2015), and by the Oxford Tropical Research Ethics Committee (OxTREC) at Oxford University (Reference number 568–15). Participants were given an information sheet and informed consent declaration, with the opportunity to ask questions, and were then asked to sign the volunteer agreement. Written consent was obtained from all participants.

### Study design

This is a mixed-methods study, with a qualitative study being conducted within a larger (quantitative) diagnostic test accuracy (DTA) study, therefore following an embedded design [37].

### Clinical specimens and specimen preparation

The recommended clinical samples for laboratory diagnosis of BU are (1) wound swabs for ulcerative lesions or (2) FNAs for non-ulcerative lesions, which include nodules, plaques or oedematous lesions. Wound swabs and FNAs were obtained between 2011 and 2015 from ulcers and non-ulcerative lesions, respectively, of patients attending clinic at the Agogo Presbyterian Hospital in the Asante Akim North Municipal (Ashanti region, Ghana) and Pakro Health Centre (PHC) in the Akwapim South district (Eastern region, Ghana). In order to increase the yield of MU cells, swabs were obtained by rounding the undermined edges of ulcerative lesions, while FNAs were taken from the weakest part of the lesions [38,39]. Swabs and FNAs were stored in 7H9 broth (Becton Dickson) supplemented with PANTA (Becton Dickson). A total of 96 clinical specimens (71 swabs and 25 FNAs) were investigated. Swabs were removed from 7H9 medium after rigorous vortexing to detach bacteria from the swabs. The suspensions were later aliquoted into microfuge tubes and stored at 4°C for DNA extraction. Samples were subjected to DNA extraction and testing as depicted in the flow chart in S1 Fig.

Sample size calculation following Hajian-Tilaki [40] was based on an assumed diagnostic sensitivity of around 90% [30], an assumed prevalence in the target population (positivity rate among those samples being sent for confirmation based on clinical screening, from the same area) of 90% [41], and a 95% confidence level of our estimates being within 0.08 of the true population value. It resulted in a minimum of 61 samples required.

### Study site

The National BU Control Programme in Ghana was established in 2000. Its aims are to minimize the morbidity and disability associated with BU disease, with the key interventions being early detection and standardized case management, reporting and surveillance. PHC, run by Ghana Health Service (GHS), was originally established in 1991. It focuses on maternal and child health/family planning and obstetric care [42] and serves inhabitants of Pakro and surrounding farming villages and hamlets. It is connected to the public electricity grid and, at the time of study conduct, did not have a generator, which means power supply could be unreliable. There were refrigerators for storage of medical supplies, which were mostly unaffected by short electricity supply cuts. Depending on the weather conditions, inside temperatures can be elevated (> 35°C). PHC is situated around 55km from Noguchi Memorial Institute for Medical Research (NMIMR), Legon, where patient samples are processed and diagnosis via PCR (as described in [30]) is established.

### DNA extraction

For comparison, two separate DNA extraction methods, namely (1) a syringe device method (SM), and (2) a conventional DNA extraction method (CM) adapted, as detailed below, from the modified Boom method [43] for the extraction of DNA from mycobacteria, were performed separately on each sample. The performance of the two extraction methods was evaluated by testing the DNA recovered in IS*2404* PCR and IS*2404* LAMP assays. As a extraction control procedure, a known concentration of MU bacilli (app. 1.0 x 10^4^ bacilli/mL) or water was included, representing positive and negative controls, respectively.

### Conventional MU DNA extraction method (CM)

DNA was extracted from swabs and FNA using the CM as described in our previous publication [30]. In a 1.5 mL microfuge tube, 150 μL of suspected BU specimen was mixed with 250 μL of lysis buffer (1.6 M GuHCl, 60 mM Tris pH 7.4, 1% Triton X-100, 60 mM EDTA, Tween-20 10%), 250 μL of glass beads, 50 μL of proteinase K. The mixtures were incubated overnight in an orbital incubator shaker (200 rpm) at 60°C overnight. The released DNA was captured by the addition of diatomaceous earth solution (prepared from 10g diatomaceous earth (Sigma Aldrich Chemi GmbH) in 50 ml of H_2_O containing 500 μl of 37% (wt/vol) HCl) and incubated in an orbital shaker at 200 rpm at 37°C. The mixtures were centrifuged at 14,000 rpm for 30 seconds and the DNA trapped unto the diatomaceous earth was washed twice with 900 μL of 70% alcohol followed by 900 μL of acetone. To remove residual acetone from DNA the mixture was evaporated by drying at 50°C for 10 min and then resuspended in 100 μL of nuclease free water and further incubated for another 10 min with intermediate mixing to release DNA trapped unto diatomaceous earth into solution. The mixture was centrifuged at 14,000 rpm for 60 seconds and the solution containing MU DNA was transferred into another microfuge tube, stored at 4°C for the IS*2404* nested PCR and IS*2404* LAMP assays.

### Syringe MU DNA extraction method (SM)

The disposable syringe device (EasyNAT, Ustar Biotechnologies (Hangzhou) Ltd, China) was originally developed for extracting *Mycobacterium tuberculosis* DNA in sputum specimens. It is operated manually and has a silica membrane inserted at the base of the syringe to adsorb and elute DNA (S2 Fig). MU DNA was extracted using SM, with modification from manufacturer’s protocol as follows: One hundred and fifty microliters of BU clinical specimen were incubated at 60°C for 30 min in 600 μL lysis buffer (1.6 M guanidine HCl, 60 mM Tris (pH 7.4), 1% Triton X-100, 60 mM EDTA, 10% Tween 20) and 50 μL Proteinase K (20 mg/mL). Lysed specimen was mixed with 1400 μL of absolute ethanol, and the tube inverted for 2–3 times to mix thoroughly. The mixture was sucked through the assembled device (aided by the plunger) and then filtered back to the tube by pushing the plunger down slowly. DNA bound to the silica membrane at the base of the device was washed twice with 2 mL of 60% ethanol using air pressure generated from pushing the plunger of the syringe. The 5 mL size syringe barrel was detached from the device and replaced with a 1 mL syringe barrel for DNA elution. Nuclease free water (100 μL) was sucked through the nozzle of the syringe (aided by the plunger) across the silica membrane 3–4 times before finally eluting in a 1.5 mL microfuge tube (S3 Fig).

### IS*2404* nested PCR

A nested PCR over two amplification rounds targeting the IS*2404* was used as the reference method for detecting MU. This method has been evaluated in other studies as reported by Stinear *et al*., [44] and Ablordey *et al*. [30,45]. IS*2404* PCR was performed on DNA recovered by both methods (CM and SM) using primers pGp1 (5’-*AGGGCAGCGCGGTGATACGG*-3’) and pGp2 (5’-*CAGTGGATTGGTGCCGATCGAG-3’)* for the first run and pGp3 (5’*-GGCGCAGATCAACTTCGCGGT-3’)* and pGp4 (5’-*CTGCGTGGTGCTTTACGCGC-3’)* for the second run. PCR for the first round was performed in a 30 μL reaction using DNeasy Blood and Tissue kit (Qiagen). The PCR master mix contains 1x PCR buffer (containing 15 mM MgCl_2_), 1X Q-solution, 0.2 mM dNTP mix, 25 pmoL each of pGp1 and pGp2, 1.0 U of HotStar Taq polymerase (Qiagen) and 3 μL of DNA extract. PCR for the second run was performed in a 25 μL master mix containing 1x PCR buffer (containing 15 mM MgCl_2_), 1X Q-solution, 0.2 mM dNTP mix, 25 pmoL each of pGp3 and pGp4, 1.0 U of HotStar Taq polymerase (Qiagen) and 1 μL of amplicons from the first run. Amplification for the first and second run was performed for 40 and 35 cycles, respectively, in a thermal cycler (T20 Thermocycler, Westburg) which was programmed at 94°C for 10 min, 94°C for 30 secs, 64°C for 60 secs, 72°C for 60 secs and a final extension at 72°C for 10 min. PCR products for the second run were electrophoresed on 2% agarose gel prepared with 1X TAE buffer stained with ethidium bromide. The size of the amplicons was estimated by comparing with 100 bp molecular weight marker (HyperLadder^TM^, Bioline) and visualized using Kodak Gel Logic 100 molecular imaging systems. Each assay was conducted with experimental controls, including a no template DNA control (NTC) and one with a known concentration of MU DNA as positive control.

### IS*2404* LAMP assay

MU DNA was amplified with the IS*2404* LAMP assay as described in our previous publication [30]. IS*2404* LAMP assays (LMP 207, Eiken Chemical, Japan) were performed on DNA recovered by CM and SM using a heat block (conventional LAMP (hbLAMP)) or pocket warmer (pwLAMP) as heat generators. LAMP was performed at 60°C for 60 min. The pwLAMP was performed by sandwiching the reaction tubes (contained in a 200 μL Eppendorf tube) in a pocket warmer (Hokaron Haru-type, Lotte Health Products, Tokyo, Japan; disposable and driven by an exothermic chemical reaction), wrapped in a paper towel and placed in a Styrofoam box for 100 min (the average time for a pocket warmer to attain 60°C is 40 min, plus 60 min of incubation) (S4 Fig). A thermocouple was sandwiched between the pocket warmer to monitor the temperature during incubation. The temperature was recorded with a DaqPRO 5300 Data logger device kindly provided by the Program for Appropriate Technology in Health (PATH) (Seattle, USA) (S5 Fig).

A total of six duplicate tests were conducted using both pocket warmer and heat block. Controls were included as for PCR above. LAMP assay results were detected visually (naked-eye detection) by observing a change in color of the reaction mixture. The initial color of the LAMP assay (orange) changes to green, indicating a positive test, but remains orange for a negative test result. The results were read within 30–60 mins post assay reaction to avoid false positives, i.e. colour change of the reaction mixture from orange to green.

### Detection limit of IS*2404* LAMP assay

An evaluation of the limit of detection (LoD) of the IS*2404* LAMP assay was performed. DNA was quantified using a Nanodrop (NanoDrop^TM^ 2000C). The mass concentration of MU DNA corresponding to 3x10^5^ copies of IS*2404* was determined based on the average genome size of 5806 kb and the average number of 207 copies of IS*2404* [46]. Serial dilution of the DNA was performed to obtain 3x10^4^, 3x10^3^, 3x10^2^, 3x10^1^ and 3 copies of the IS*2404* target, and each used as template for the IS*2404* LAMP assay.

### Statistical analysis

Statistical significance between two assays was determined using SPSS version 18.0 [47]. A test was considered significant if the *p*-value < 0.05. The Kappa coefficient (κ) was used to compare the level of agreement between LAMP assays [48]. Assay sensitivity, specificity, positive predictive value (PPV) and negative predictive value (NPV) were calculated using epi.tests in R 4.3.1 [49]. Sample size was calculated using epi.ssdxsesp in R 4.3.1 [49], based on the method proposed by Hajian-Tilaki [40].

### Conduct of interviews and FGD

Individual semi-structured interviews (IIs) and one FGD were conducted between 2015 and 2016. Using a purposive convenience sampling approach with snowball sampling, where recruited participants recommended additional ones [50], participants were selected based on (1) their role in the diagnosis and treatment of BU at PHC and (2) their role as researchers in the evaluation study. We conducted nine IIs with two researchers (R1 and R2), two health care professionals (HCPs) involved in diagnosis and treatment of BU at PHC (H1 and H2), and five community-based surveillance (CBS) volunteers (C1-C5). In addition, we conducted one FGD at PHC with two researchers (R1 and R2) and two HCPs treating patients at PHC (H1 and H2). All participants also remained available for several short, informal follow-up interviews. The approximate duration of an II or FGD was one hour, with refreshments provided. The language was English, with the help of a translator if necessary. Before study onset, study objectives were thoroughly explained to each participant. The topic guide used for the IIs included the process of diagnosis, training, study logistics, perceived gaps in knowledge or understanding, either subjective or actual gaps in evidence, and barriers (either experienced or expected). For the FGD, topic areas covered the process of diagnosis and treatment, as well as experienced barriers. One researcher (ACE) conducted the IIs and FGD and audio recorded the discussions. Recordings were transcribed, verbatim, and transcriptions were proofread repeatedly against the audio file to check for accuracy, in order to add missing information. The researcher took detailed notes before, during and after the interviews as well as regarding observations made at the study site during repeated visits.

### Analysis of interviews and FGD

Transcripts were coded using NVivo 11 [51], and analysed using a thematic analysis approach [52]. Themes are concepts that describe, help to interpret and explain aspects of the data [53]. Examples for themes relevant to this study include timelines or specific barriers to diagnosis and follow-up. Transcription and preliminary thematic analysis for initial interviews were performed in order to inform subsequent interviews, incorporating emerging themes. The analysis was informed by the notes taken.

## Results

### Evaluation of the syringe method (SM) for DNA extraction

**Fig 1:**
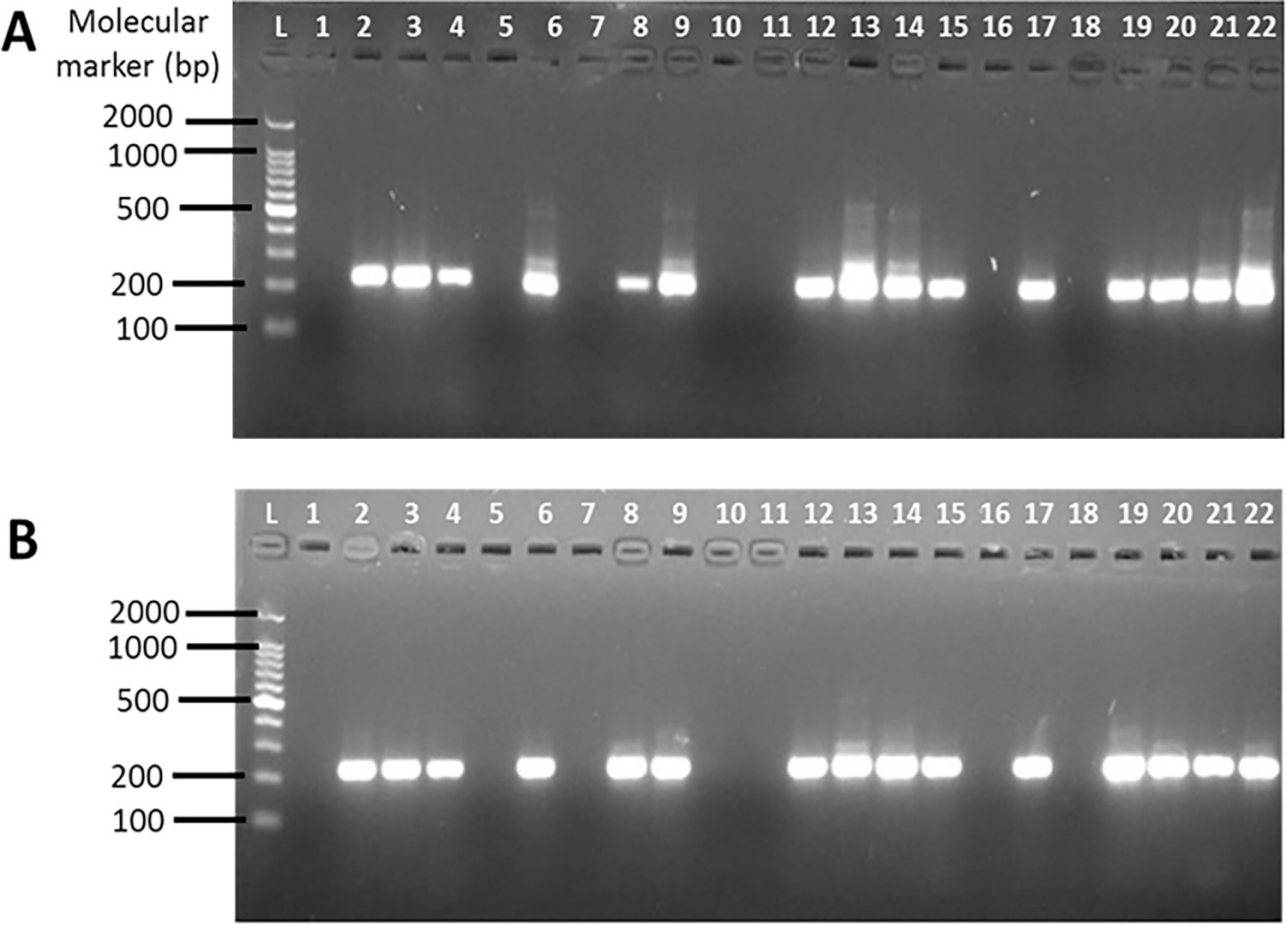
Detection of MU DNA by IS*2404* nested PCR. A subset of 20 DNA extracts (selected randomly) from the total 96 DNA extracts obtained by CM (A) and SM (B). DNA extracts were amplified by IS*2404* PCR. Amplicons (5μL) were analysed on 2% agarose gel stained with ethidium bromide in 1X TAE buffer. Lanes 1 and 2 in both (A) and (B) corresponds to negative and positive extraction and amplification controls, respectively, representing the absence or presence of MU bacilli from culture in the DNA extraction procedure and PCR. Lanes 3–22 in (A) and (B) are samples amplified using DNA extracted by CM and SM, respectively. No DNA band represent a negative test.

IS*2404* PCR^CM^ was considered as the reference method. Of a total of 96 clinical specimens, 81 tested positive (84.4%) and 15 tested negative (15.6%) for MU by IS*2404* PCR^CM^ (reference method). IS*2404* PCR using SM DNA extracts (IS*2404* PCR^SM^) failed to detect 8 MU cases that tested positive with the reference method (Table 1), resulting in a sensitivity of 90.12% (95% CI, 81.46–95.63) and a specificity of 100% (95% CI, 78–100) of IS*2404* PCR^SM^ as compared to IS*2404* PCR^CM^. The positive predictive value (PPV) and the negative predictive value (NPV) for IS*2404* PCR^SM^ were determined at 100% (95% CI, 95.07–100) and 65.22% (95% CI, 42.73–83.62), respectively. In comparison, there was good agreement between the reference method and IS*2404* PCR^SM^ as determined by the Cohens Kappa coefficient (κ = 0.74). The gel electrophoretogram (Fig 1) shows a representation (20 out of 96) of the total DNA amplified.

**Table 1:** Comparison of sensitivity and specificity of IS*2404* PCR with DNA extracts obtained by CM and SM from a total of 96 BU clinical specimens.

	Conventional Method (CM)			
		+	-	Total
	+	73	0	73
**Syringe Method (SM)**	-	8	15	23
	Total	81	15	96
**% Sensitivity**	90.12			
**% Specificity**	100			

IS*2404* PCR sensitivity and specificity of SM in comparison to CM (recommended method of BU testing). (+) and (-) refers to positive and negative tests, respectively.

We also compared the suitability of the specimen type (swab and FNA) for DNA extraction. Out of the 71 FNA specimens tested, 65 (91.5%) were MU positive by IS*2404* PCR^CM^ and 61 (85%) were IS*2404* positive by IS*2404* PCR^SM^. The difference was not statistically significant (*p*>0.05). With 25 swabs tested, 16 (64%) and 12 (48%) recovered by the CM and S, respectively, were IS*2404* positive. The difference was not statistically significant (*p*>0.05). Thus, 4 samples each from FNA and swabs respectively were not detected by IS*2404* PCR^SM^.

### Evaluation of the pocket warmer (pw) LAMP for DNA amplification

Sixty-four clinical specimens (45 FNA and 19 swabs) were randomly selected out of the 96, consisting of 56 positives and 8 negatives previously determined by IS*2404* PCR. The corresponding DNA extracts, using both CM and SM, were subjected to the IS*2404* LAMP assay, using a pocket warmer (pwLAMP) or a heat block (hbLAMP) for incubation. With the hbLAMP^CM^ or hbLAMP^SM^ assay, a positivity rate of 84.4% and 71.9%, respectively, was obtained (Table 2). The sensitivity, specificity, PPV and NPV of the pwLAMP^SM^ was 83.6%, 100%, 100% and 50%, respectively. Based on the Kappa coefficient value, the level of agreement between pwLAMP^SM^ and IS*2404* PCR^CM^ was considered as good (κ = 0.64). There was no difference in the positivity rate and sensitivity between hbLAMP^SM^ and pwLAMP^SM^(Table 2). It was observed that the pocket warmer required 40 min to reach 60°C, and was subsequently able to provide the required temperature needed for 60 min incubation (S5 Fig).

**Table 2:** Analysis of CM and SM DNA extracts using LAMP assay and IS*2404* PCR.

	**hbLAMP** ^**CM**^			**IS*2404* PCR** ^**SM**^			**hbLAMP** ^**SM**^			**pwLAMP** ^**SM**^		
	+	-	Total	+	-	Total	+	-	Total	+	-	Total
**IS*2404* PCR**^**CM**^ **(+)**	54	2	56	49	6	55	46	9	55	46	9	55
**IS*2404* PCR**^**CM**^ **(-)**	0	8	8	0	9	9	0	9	9	0	9	9
**Total**	54	10	64	49	15	64	46	18	64	46	18	64
**% Positivity**	84.4			76.6						71.9		
**% Sensitivity**	96.4			89.1						83.6		

IS*2404* PCR^CM^ = IS*2404* PCR performed using DNA recovered by the CM

hbLAMP^CM^ = conventional LAMP assay performed using DNA recovered by the CM

IS*2404* PCR^SM^ = IS*2404* PCR performed using DNA recovered by the SM

hbLAMP^SM^ = conventional LAMP assay performed using DNA recovered by the SM

pwLAMP^SM^ = pocket warmer assay performed using DNA recovered by the SM

### Evaluation of the LoD

**Fig 2:**
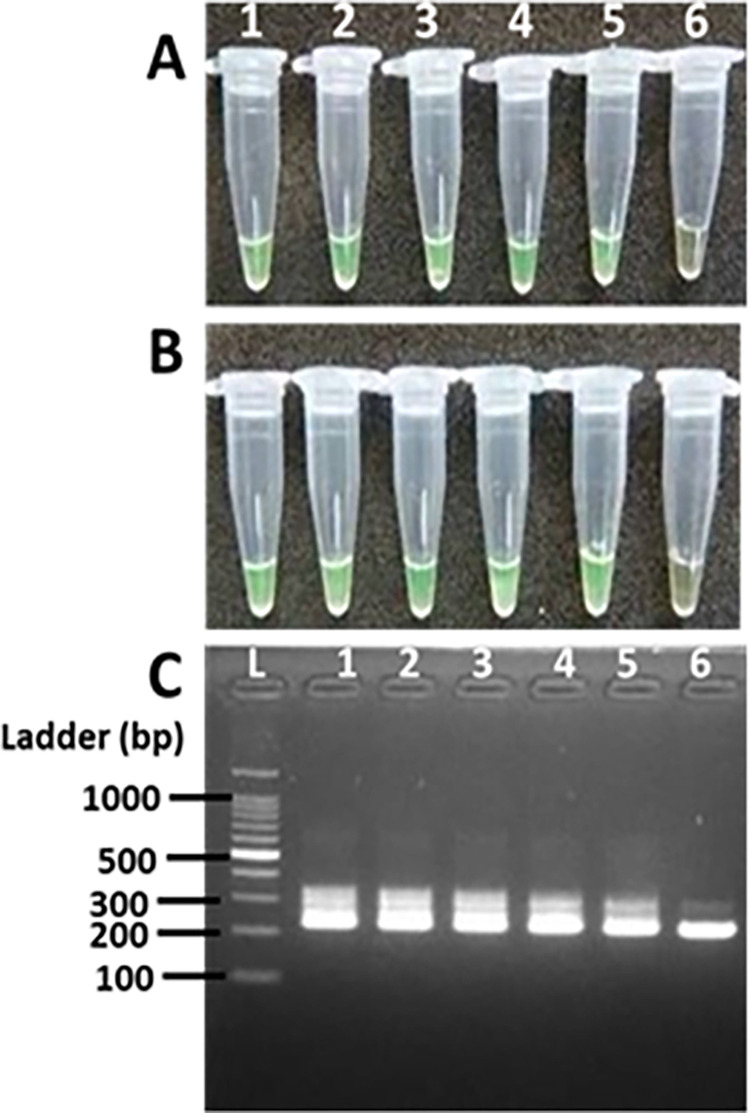
Comparing sensitivity of the cLAMP (A), pwLAMP (B), and IS*2404* PCR (C) using varying concentrations of MU DNA. Tube 1–6 represent 3x10^5^, 3x10^4^, 3x10^3^, 3x10^2^, 3x10^1^ and 3 copies of IS*2404*, respectively. Lane L: 100 bp molecular ladder; lanes 1–6 contains 3x10^5^, 3x10^4^, 3x10^3^, 3x10^2^, 3x10^1^ and 3 copies of IS*2404* respectively.

We further tested the analytical sensitivity (LoD) of the LAMP assay. Since the LAMP assay targets and amplifies the IS*2404*, we calculated the number of this target present in dilutions of MU DNA extracted by the SM. Based on our calculations [46], the diluted DNA contained approximately 3x10^5^, 3x10^4^, 3x10^3^, 3x10^2^, 3x10^1^ and 3 copies of IS*2404*. LAMP assay was tested on each dilution. The hbLAMP and pwLAMP detected to the limit of 30 copies of the IS*2404*, while IS*2404* PCR detected 3 copies of IS*2404* (Fig 2).

### BU diagnosis and treatment at Pakro Health Centre

Interviews and FGD yielded a rich picture of the diagnostic and treatment workflow at PHC, complementing the technical assessment presented above, and exploring a potential target setting. In the following, findings are presented, with quotes are typeset in italics; abbreviations correspond to respondents’ ID.

BU was often perceived as a non-medical condition; therefore, alternative treatment (herbal or spiritual) was sought first, delaying the onset of medical treatment. Sensitization and education activities were seen as crucial, and have already led to a change in attitudes.

*As [patients] perceive [Buruli] as witchcraft, […] they visit a whole lot of herbalists before coming, that is why they are coming late with the bad ulcers. (H1)*

Patients present at PHC, usually on Thursdays, which are clinic days. HCPs ask about patients’ disease history and take samples for BU diagnosis. BU diagnosis based on clinical criteria was considered very accurate in general, so that a large proportion of suspected patients would later be confirmed to be BU positive.

As per an agreement with GHS, samples from PHC were sent directly to the laboratory at NMIMR, in order to shorten the time to diagnosis. Samples were taken from Pakro to the bus station in Nsawam, usually on a motorbike or by shared taxis. The samples were then transported by public minibuses to Madina in Accra, their terminal station, covering a total distance of around 55 km.

*[We take] the swabs and [complete] the forms [provided by the National BU Control Programme], and send them to the station, then give them to the bus drivers. […]. And we call Noguchi [researchers at NMIMR), so they go to the station to meet the drivers and collect the samples from them. […] We give the numbers of the vehicles, the driver’s name, the driver’s number, and then they call them, meet them there [in Madina], and […] pay some token to them [for] transport. (H2)*

In the absence of such an arrangement, samples would be sent to the district office, and via the regional office then onwards to one of the three reference laboratories in Ghana, of which Dr. Ablordey’s lab at the NMIMR is one.

*But in more remote places then the timeline would just be much much much longer. (H2)*

The time delay from taking the samples for confirmation of diagnosis was from 48 hours to a maximum of around two weeks, with a typical time of about one week. The diagnosis was confirmed to the HCP via e-mail or text message, and then the HCP informed the patient via a mobile phone number (which could be a relative’s or neighbour’s number, or the number of a CBS volunteer). Due to time and financial constraints, it then took on average three days to one week until patients presented at the clinic for treatment, consisting of oral rifampicin and streptomycin injections for 8 weeks, accompanied by wound dressing, initially requiring daily visits. During that time, the wounds were dressed, and often, trained CBS volunteers helped with wound dressing. Wounds of patients with a negative diagnosis were also managed appropriately.

A recurring theme was the problematic transport situation for patients to and from the facility, via shared means of transport, for example during market days. One CBS volunteer gave an example: His community is about 3.5 miles from Pakro along the road. It’s a scattered community of farmers, and produce is being sold, mostly by women, on the local markets on specific market days. On these days, transport options for patients are available: Monday and Thursday, on market days in Nsawam, large public buses (Metro Mass Transit, or MMT) run along the main road in the morning and evening to transport passengers and goods to and from the market. On Tuesday, market day in Adonso, (shared) taxis are usually available. On other days, no public transport options are available. Sometimes motorbikes take people between villages, but *‘It’s just off/on*, *it’s not always that you get it’ (C2)*. The standard shared taxi fare from the village to Pakro was two Ghana Cedis, corresponding to the price of a meal with meat at a street kitchen.

As a recurring motive, availability of funds for transport was brought up repeatedly.

*(When patients hear about their diagnosis,) some of them don’t feel happy. […] We’ll go to the communities, we’ll get them, and then they’ll promise they’ll come, but they will not come. Some […] don’t finish the antibiotic treatment […]. When you call and you ask, they say ‘financial problem’. It’s bad. (H1)*

*We have patients coming here [from remoter areas] because they hear of this place […]*. *And we have a challenge of keeping these patients*. *(H2)*

Alternatively, for example if patients are very old, health care staff will administer treatments.

*And the very old ones, sometimes they cannot walk, […] because of the BU. So […] the technical staff take their motorbike, go there, give the treatment, dress their wounds, and then they would come back. (H2)*

Volunteers mentioned mobility challenges, that is, problems reaching families in scattered villages for home visits, in particular during the rainy season. They would visit after dark, when families were back from farming activities, and it’s only small footpaths connecting the hamlets. They indicated the need for raincoats, umbrellas and wellington boots, torchlights, and repair for bicycles.

It was suggested that treatment is administered in health compounds closer to home villages, if there is one.

*If there’s a health facility closer to the patients’ home, the medication will be given to the patients directly, with a note attached to it, so they can be treated daily at the other facility and will visit Pakro for check-up: after several weeks they come again for refilling. (H2)*

In summary, timely diagnosis of BU is of importance in order to ensure efficient treatment, and in order to prevent patients from being lost to follow-up. In addition to reliable screening based on clinical assessment, a diagnostic method used for preliminary confirmation of BU at the PoC, adapted to the local conditions, would be seen as an advantage.

## Discussion

The demand for a rapid and accurate test for BU diagnosis at the community or primary health-care level, at the PoC, has been emphasized during a consensus meeting convened by the Foundation for Innovative New Diagnostics (FIND) and World Health Organization/Neglected Tropical Diseases (WHO/NTD) on BU diagnosis in 2018 [54]. It is also among the research priorities of WHO’s Global BU Initiative [20] and WHO’s 2021–2030 NTDs road map [55].

Nucleic-acid based detection techniques for diagnosis of BU are considered ideal [36], but require efficient and robust protocols capable of extracting MU DNA from clinical samples, as well as methods for DNA amplification. Our study addresses this need by evaluating pwLAMP^SM^, a DNA-detection-based protocol that does not require constant electricity supply for those two components, DNA extraction and -amplification. We complemented this evaluation study of diagnostic test accuracy by characterization of the diagnostic and treatment workflow at the research site in Pakro, Ghana, a primary-level health care facility in a BU endemic area, where the time to diagnostic confirmation of BU is a challenge.

In line with previous reports from endemic areas in Ghana, Benin and Cameroon [56–60], we found that BU was often perceived as a spiritual condition, with treatment being sought from traditional healers first. This could be related to the as-of-yet unclear mode of transmission of BU in West Africa [61]. Affected persons were found to delay treatment-seeking due to financial constraints and the absence of pain, as discussed previously [58,62]. Delayed treatment in turn leads to disease progression, and more complex treatment regimens [3]. In our study, the role of community-based volunteer health workers was emphasized; their role has been further investigated in a recent study conducted in the Ashanti region of Ghana, underlining their importance in complementing government efforts to improve resource availability for healthcare services [63]. We describe an example of the diagnostic workflow at PHC; the typical estimated delay between a sample being taken and the results being communicated was 48 hours to around one week. This potentially exceeds estimates reported from a study in Ghana including 75 healthcare facilities in seven regions, including our study area, from the same time period [64]. Its authors reported an average of 24 hours for sample transport alone; however, they did not indicate differences between major and peripheral health facilities, and between individual ones, and the role of courier services.

Screening based on the attending clinician’s diagnosis was seen as very accurate, corresponding to previous reports of a sensitivity greater than 90% [41,65]. In contrast, a study from Ghana found the positivity rate among those samples being sent for confirmation based on clinical screening to be only 50% [64], indicating considerable differences between areas or study sites. The high accuracy of assessment based on clinical criteria has been considered in in the development of a ‘Buruli Score’, a multivariable prediction model for diagnosis of BU in individuals with ulcerative skin lesions in Cameroon [66]. and in WHO’s updated BU clinical reporting forms, released in 2020 [67]. In line with this, stakeholders for a WHO consultation process agreed that clinical assessment has an acceptable PPV and therefore, the need for developing screening tests at the community level was not given priority [54].

The current WHO target for recommended confirmation with quantitative real-time PCR (qPCR), requiring adequately equipped laboratory facilities, was not being met as of 2018 [68]. The establishment of a new BU-LABNET in African countries has sought to improve the infrastructure for laboratory confirmation [68]; furthermore, BU is integrated into WHO’s 2021–2030 roadmap for NTDs [55], with a laboratory confirmation rate of 95% as key indicator.

A field-reliable DNA extraction method as well as provision of isothermal conditions for amplification independent of electricity supply remain a major challenge towards developing LAMP as a DNA-detection based test for field application [69,70]. Extraction of MU DNA from clinical samples typically requires the use of electricity-driven centrifuges. This study sought to address this by evaluating a simple handheld syringe device for extraction of MU DNA. The lower sensitivity of *IS2404* PCR^SM^, as compared to *IS2404* PCR^CM^, is likely due to the less efficient DNA extraction procedure, as the use of glass beads and lengthy incubation time (12 hours) required for the CM might have resulted in a much more efficient cell lysis and the release of significant amount of MU DNA. Mycobacteria species in general have thick cell walls mainly composed of mycolic acids that are resistant to chemical lysis [71,72]. In addition, the application of centrifugation steps enhances DNA yield [71,72]. Thus, the absence of rigorous cell lysis and centrifugation procedures which are key steps in DNA extraction were omitted in our SM and this may have contributed to the reduced DNA yield. In addition, possible DNA fragmentation resulting from the upward and downward thrust of the syringe plunger could have compromised DNA integrity. Since the SM does not require the use of centrifuge, it takes approximately 45 mins to complete DNA extraction and 100 mins for IS*2404* LAMP assay, thus a total of 150 mins is required, less than the 240 mins required for IS*2404* nested PCR. Beissner *et al*. [32] focused on overcoming the cold chain requirement for BU diagnosis in remote areas. 140 clinical samples from 91 suspected Buruli ulcer disease patients were tested with IS2404 dry-reagent-based (DRB) PCR, conventional IS2404 PCR (cPCR), and IS2404 qPCR, in comparison to conventional LAMP (cLAMP) assay. As compared to qPCR, the sensitivity of DRB-PCR and cPCR was determined to be 86.76%, and of cLAMP 83.82%. On a set of a further 24 samples, tested positive in all routine assays, sensitivity of cLAMP (95.83%) and DRB-LAMP (91.67%) were found to be comparable.

LAMP has been reported as a promising tool for the diagnosis of BU under field conditions [30–32]. The LAMP protocol evaluated here fulfils a number of (at least minimum) requirements of the TPP of a rapid test for diagnosis of BU, developed by WHO and FIND [36]. Requirements include DNA as target to be detected, with results being available the same day (and, with a total duration of 150 mins, faster than IS*2404* nested PCR). Sample types used are lesion swabs or FNA; visual (naked-eye) endpoint detection, both stipulated among the ideal requirements in the TPP, is possible for the LAMP protocols evaluated here. Although the TPP specifies ‘battery-operated’ among the power requirements, we would argue that any independence of a constant electricity supply should be acceptable: pwLAMP^SM^ presented and evaluated here would fit this criterion; alternative heat sources that have been tested are water baths [34] or the NINA [70]. 3–5 steps for sample preparation are considered acceptable, with the total number of steps to be performed by operator limited to less than 10, with one timed step. Currently, pwLAMP^SM^ exceeds this limit.

The formulation of an optimized lysis buffer with efficient lytic effect of mycobacterial cells and minimal effect on the MU DNA is needed. Further research activities should aim at evaluating the protocol directly at the PoC and/or other sites, including staff training requirements, testing of lyophilized reaction mixtures, assessing its sensitivity to elevated temperatures and to the risk of amplicon contamination [73]; as well as testing its specificity using non-African MU strains. Such a study should include a larger sample size that would permit e.g., stratification by sample and patient type, as well as other modifications to the protocol, and an analysis of price in comparison to other tests.

Since the time this study was conducted, a new recommended treatment regimen, an all-oral combination of rifampicin and clarithromycin [74], has become available, in addition to complementary treatments such as wound care. This combination therapy has a safety profile superior to the drug combination of rifampicin (10 mg/kg orally once daily) and streptomycin (15 mg/kg intramuscularly once daily) for 8 weeks, the standard treatment recommendation at the time of study conduct, with considerable nephro- and ototoxicity and the requirement for a daily injection [3]. From a diagnostic perspective, this could also be considered a reduction in the risk related to treatment based on a false-positive diagnosis [75]. Lastly, other field-applicable DNA-detection based methods for diagnosis of BU have become available and are being evaluated, such as Biomeme PCR [76].

### Strengths and limitations of this study

This exploratory study evaluates an electricity-free protocol for DNA-based detection of MU. It combines a diagnostic test accuracy (DTA) study with qualitative methodology to provide a picture of a clinic in a BU-endemic area as a potential target setting for diagnosis at the community or primary health care level. Methods used, such as the reference standard IS*2404* nested PCR, reflect the technical equipment in the study setting during the study period (2014–2018). Technically superior alternatives, already implemented in other settings during our study period, include qPCR, with an increased analytic and diagnostic sensitivity as compared to PCR [77,78] but higher demands in terms of equipment. Our panel did not include samples from patients suffering from skin conditions similar to BU as negative controls, and it was not possible to carry out further experiments after study closure. For the qualitative part, generalizability of findings was not aimed at, and the number of participants was determined accordingly [79]. Lastly, we did not consider costs when assessing the protocol’s suitability as a diagnostic tool for the given context, as materials were mostly provided at reduced prices, or donated, for use in this research.

## Conclusion

Our study demonstrated the diagnostic accuracy and further optimization needs of LAMP^SM^ in relation to its potential use for diagnosis of BU in a community-based primary health care setting. DNA recovery from BU clinical specimens using this simple syringe-based device could be performed in low-resource endemic areas where specific laboratory equipment (centrifuges) and constant electricity supply are not available, saving time and requiring minimal expertise as compared to conventional methods. The observation that hbLAMP^SM^ and pwLAMP^SM^ assays yielded concordant results when tested on the same set of clinical samples further gives an indication of the potential suitability of the pocket warmer in providing isothermal incubation.

## Supporting information

S1 Fig Flow chart of assay procedure (sampling, DNA extraction and DNA testing). Two separate DNA extractions (modified Boom method (CM) and syringe method (SM)) was performed on all 96 suspected BU clinical specimens. Out of the total DNA extracts (n = 96), 64 each were selected tested by IS2404 PCR or LAMP assays. All DNA tests were performed in duplicate and were compared to the gold standard (IS2404 PCRBM).
(DOCX)

S2 Fig Disposable syringe device. The syringe device is composed of a 5mL syringe barrel fitted to the syringe nozzle with a silica membrane embedded at the base.
(DOCX)

S3 Fig Schematic representation of DNA extraction procedure using syringe device. (A) Lysed sample sucked through the assembled silica syringe device (B) Washing of the DNA bound to the silica membrane in assembled syringe device (C) Elution of DNA from bound silica membrane with a 2 ml syringe.
(DOCX)

S4 Fig Illustration of the pocket warmer LAMP assay. (A) Pocket warmer providing isothermal conditions for the LAMP reaction. Arrow indicating pocket warmer. (B) PCR tubes containing LAMP reaction arranged in pocket warmer. (C) Pocket warmer wrapped over reaction tubes and (D) Pocket warmer placed in Styrofoam box for incubation.
(DOCX)

S5 Fig Temperature profile of a pocket warmer. Temperature generated by the pocket warmer was monitored using a thermocouple sandwiched between the pocket warmer. Temperature readings were obtained in duplicate on two separate days. Error bars indicate standard deviation of the average readings.
(DOC)

S1 Data Raw data file of Supporting information- OMEGA OM-DAQPRO-5300 to monitor temperature profile of pocket warmer. (XLSX)
