# Supplementary material for: Evaluation of an electricity-independent method for IS2404 Loop-mediated isothermal amplification (LAMP) diagnosis of Buruli ulcer in resource-limited settings
Source: PLoS Negl Trop Dis. 2024 Aug 14;18(8):e0012338. doi: 10.1371/journal.pntd.0012338 (PMC11346967; doi:10.1371/journal.pntd.0012338)
Supplement: S1 Fig — Two separate DNA extractions (modified Boom method (CM) and syringe method (SM)) was performed on all 96 suspected BU clinical specimens. Out of the total DNA extracts (n = 96), 64 each were selected tested by IS2404 PCR or LAMP assays. All DNA tests were performed in duplicate and were compared to the gold standard (IS2404 PCRBM). (DOCX) [file pntd.0012338.s001.docx]

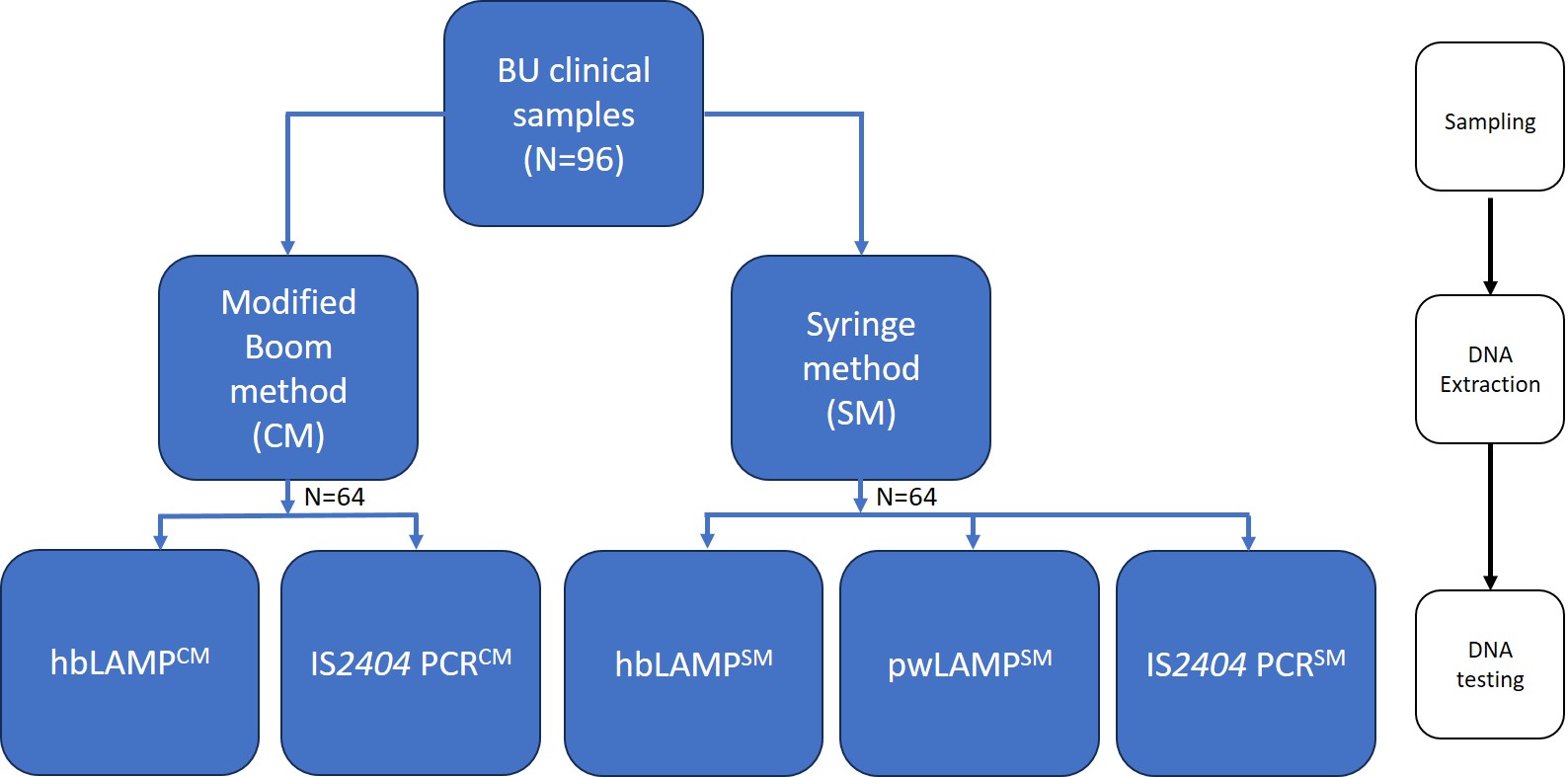


**S1 Fig. Flow chart of assay procedure (sampling, DNA extraction and DNA testing)**. Two separate DNA extractions (modified Boom method (CM) and syringe method (SM)) was performed on all 96 suspected BU clinical specimens. Out of the total DNA extracts (n=96), 64 each were selected tested by IS*2404* PCR or LAMP assays. All DNA tests were performed in duplicate and were compared to the gold standard (IS2*404* PCR^CM^).
