# Supplementary material for: Evaluation of an electricity-independent method for IS2404 Loop-mediated isothermal amplification (LAMP) diagnosis of Buruli ulcer in resource-limited settings
Source: PLoS Negl Trop Dis. 2024 Aug 14;18(8):e0012338. doi: 10.1371/journal.pntd.0012338 (PMC11346967; doi:10.1371/journal.pntd.0012338)
Supplement: S2 Fig — The syringe device is composed of a 5mL syringe barrel fitted to the syringe nozzle with a silica membrane embedded at the base. (DOCX) [file pntd.0012338.s002.docx]

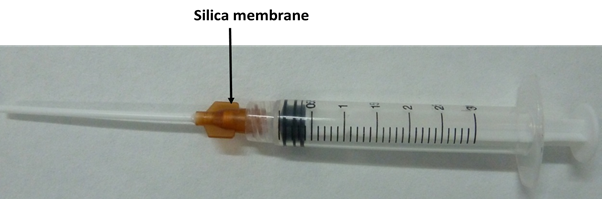


**S2 Fig. Disposable syringe device**. The syringe device is composed of a 5mL syringe barrel fitted to the syringe nozzle with a silica membrane embedded at the base.
