# Supplementary material for: Evaluation of an electricity-independent method for IS2404 Loop-mediated isothermal amplification (LAMP) diagnosis of Buruli ulcer in resource-limited settings
Source: PLoS Negl Trop Dis. 2024 Aug 14;18(8):e0012338. doi: 10.1371/journal.pntd.0012338 (PMC11346967; doi:10.1371/journal.pntd.0012338)
Supplement: S3 Fig — (A) Lysed sample sucked through the assembled silica syringe device (B) Washing of the DNA bound to the silica membrane in assembled syringe device (C) Elution of DNA from bound silica membrane with a 2 ml syringe. (DOCX) [file pntd.0012338.s003.docx]

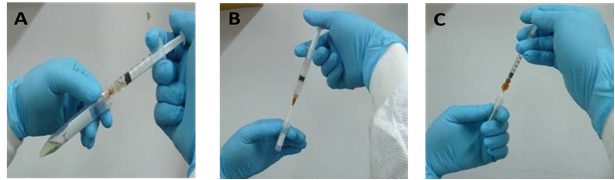


**S3 Fig. Schematic representation of DNA extraction procedure using disposable silica syringe device**. (A) Lysed sample sucked through the assembled silica syringe device (B) Washing of the DNA bound to the silica membrane in assembled syringe device (C) Elution of DNA from bound silica membrane with a 2 ml syringe.
