# Supplementary material for: Evaluation of an electricity-independent method for IS2404 Loop-mediated isothermal amplification (LAMP) diagnosis of Buruli ulcer in resource-limited settings
Source: PLoS Negl Trop Dis. 2024 Aug 14;18(8):e0012338. doi: 10.1371/journal.pntd.0012338 (PMC11346967; doi:10.1371/journal.pntd.0012338)
Supplement: S4 Fig — (A) Pocket warmer providing isothermal conditions for the LAMP reaction. Arrow indicating pocket warmer. (B) PCR tubes containing LAMP reaction arranged in pocket warmer. (C) Pocket warmer wrapped over reaction tubes and (D) Pocket warmer placed in Styrofoam box for incubation. (DOCX) [file pntd.0012338.s004.docx]

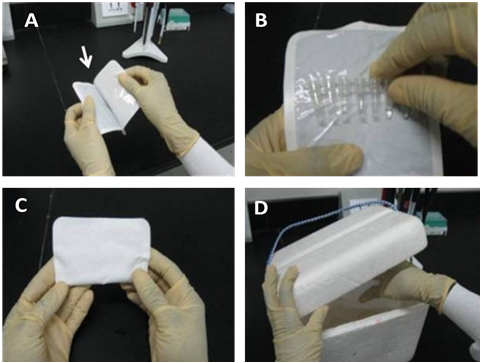


**S4 Fig. Illustration of the pocket warmer LAMP assay**. (A) Pocket warmer providing isothermal condition for LAMP reaction. Arrow indicating pocket warmer (B) PCR tubes containing LAMP reaction arranged in pocket warmer (C) pocket warmer wrapped over reaction tubes and (D) pocket warmer placed in Styrofoam box for incubation.
