# Supplementary material for: Evaluation of an electricity-independent method for IS2404 Loop-mediated isothermal amplification (LAMP) diagnosis of Buruli ulcer in resource-limited settings
Source: PLoS Negl Trop Dis. 2024 Aug 14;18(8):e0012338. doi: 10.1371/journal.pntd.0012338 (PMC11346967; doi:10.1371/journal.pntd.0012338)
Supplement: S5 Fig — Temperature generated by the pocket warmer was monitored using a thermocouple sandwiched between the pocket warmer. Temperature readings were obtained in duplicate on two separate days. Error bars indicate standard deviation of the average readings. (DOC) [file pntd.0012338.s005.doc]

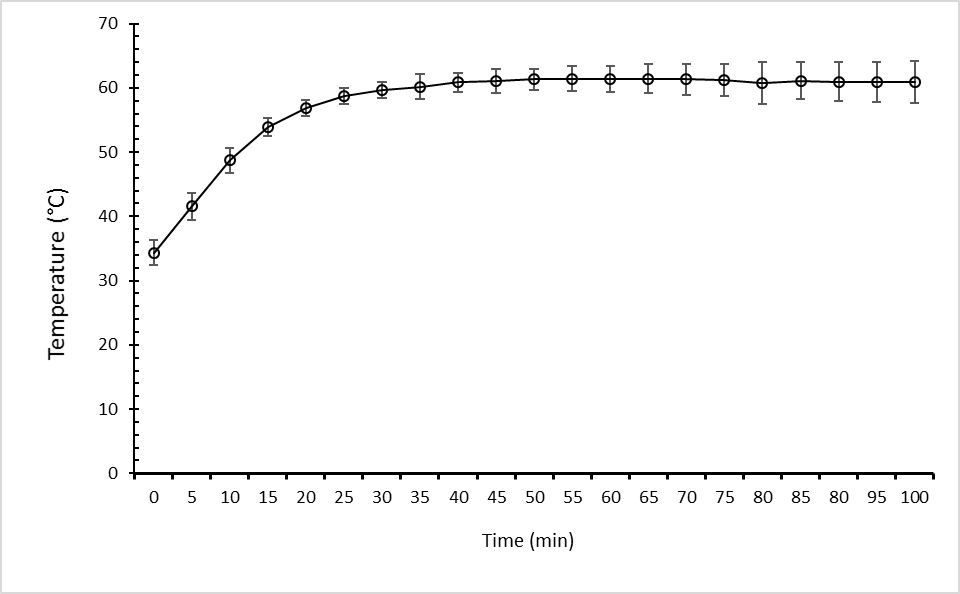


**S5 Fig. Temperature profile of a pocket warmer**. Temperature generated by the pocket warmer was monitored using a thermocouple sandwiched between the pocket warmer. Temperature readings were obtained in duplicate on two separate days. Error bars indicate standard deviation of the average readings.
